# Supplementary figures and images for: Tripartite interactions between filamentous Pf4 bacteriophage, Pseudomonas aeruginosa, and bacterivorous nematodes
Source: PLoS Pathog. 2023 Feb 17;19(2):e1010925. doi: 10.1371/journal.ppat.1010925 (PMC9980816; doi:10.1371/journal.ppat.1010925)

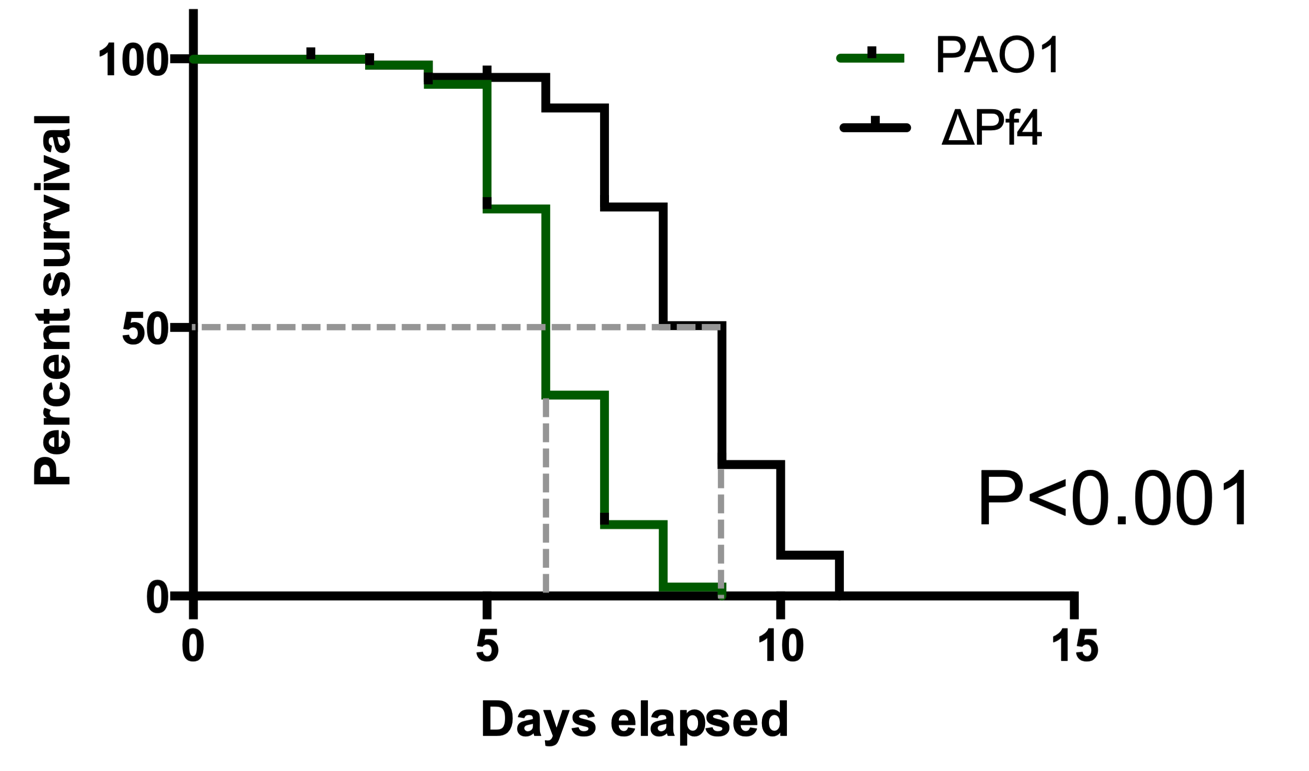

Supplement: S1 Fig — Kaplan–Meier survival analysis of N = 90 worms per condition (three replicate experiments of 30 worms each) were monitored daily for death. The mean survival of rrf-3(-); fem-1(-) C. elegans maintained on lawns of PAO1 was six days compared to nine days for nematodes maintained on lawns of ΔPf4 (dashed gray lines). (TIFF) [file ppat.1010925.s001.tiff]

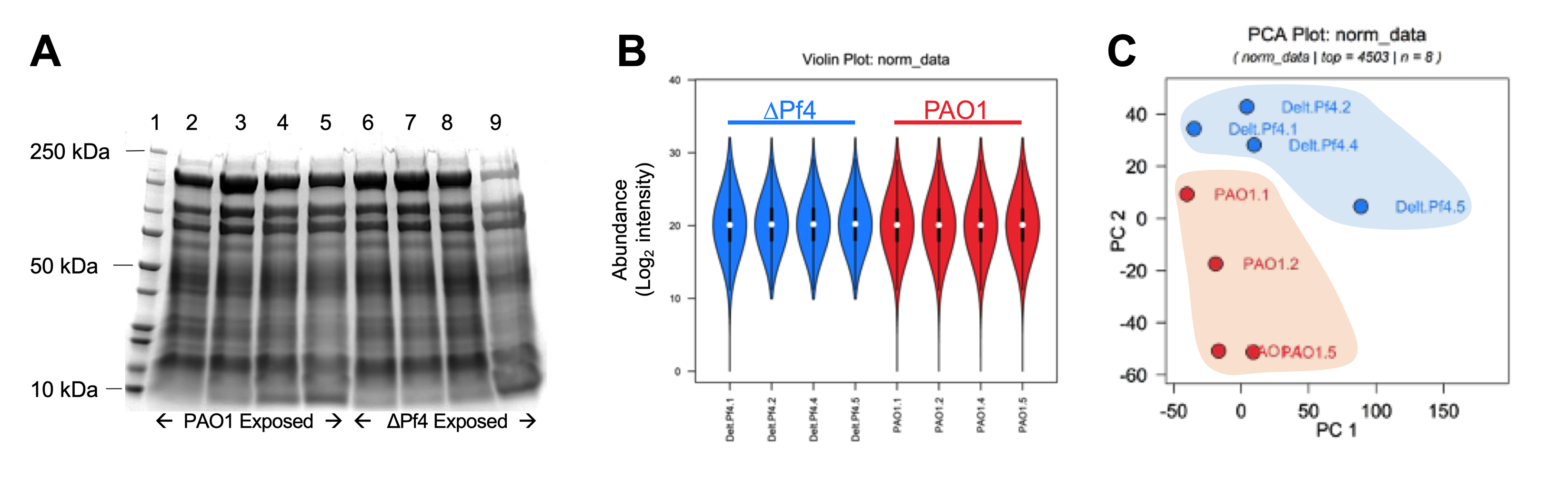

Supplement: S2 Fig — (A) C. elegans exposed to PAO1 or ΔPf4 show similar total protein profiles. Forty-five μg of total protein extracted from C. elegans rrf-3(-); fem-1(-) exposed to either PAO1 or ΔPf4 for 48 hours was loaded onto a 4–15% Tris Glycine SDS gel and stained with Coomassie blue. Lane 1 Precision Plus All Blue Standard (Bio-Rad 1610373), Lanes 2–5 biological replicates of PAO1 exposed C. elegans, Lanes 6–9 ΔPf4 exposed C. elegans. Note that after sufficient protein was set aside for mass spectrometry analysis, protein for the sample in lane 9 was limiting, so less was loaded (~35 μg/μL). (B) Log2 transformed peptide intensity values were comparable in all datasets. (C) Principal component analysis (PCA) shows that biological replicates cluster within groups. (TIFF) [file ppat.1010925.s002.tiff]
