# Supplementary material for: Tripartite interactions between filamentous Pf4 bacteriophage, Pseudomonas aeruginosa, and bacterivorous nematodes
Source: PLoS Pathog. 2023 Feb 17;19(2):e1010925. doi: 10.1371/journal.ppat.1010925 (PMC9980816; doi:10.1371/journal.ppat.1010925)
Supplement: Attachment — Submitted filename: PLOS Pathogens Reviewer comments.pdf [file ppat.1010925.s004.pdf]

Dear Editor,
Thank you for handling the review of this manuscript. We genuinely appreciate the feedback, and we think our response to the reviewer's suggestions has improved the work. The following is a point-by-point response to each concern. We hope the revised manuscript makes a scientific contribution consistent with the reputation of *PLOS Pathogens*.

Please note that the line numbers indicated below refer to the track changes document.

Part I - Summary

Reviewer #1: Review of Tripartite interaction between filamentous Pf4 bacteriophage, Pseudomonas aeruginosa, and bacterivorous nematodes.

Schwartzkopf and colleagues have written a well structed study into the interaction between QS Pf4 and C elegans and PAO1. We are presented the observation that Pf4 cured strains (∆Pf4) are less virulent than wildtype even though they typically produce higher levels of a known virulence factor pyocyanin. This counter intuitive claim is followed by an examination of the model (c elegans) immune response to virulence factors. Overall, I think this paper should be published and the findings are of broad interests to several disciplines. I have some minor comments which I think would improve the paper and a couple of minor mistakes which need addressing.

Reviewer #2: In this work, Schwartzkopf et al. demonstrate mechanisms by which bacteriophage Pf4 influences Pseudomonas aeruginosa virulence. Using C. elegans as a model, they show that WT PAO1 is more virulent than strains that have been cured of their Pf4 infection (DPf4), despite DPf4 overproduction of pyocyanin. They demonstrate mutant C. elegans with impaired pigment detection are more susceptible to DPf4, suggesting a mechanism of resistance in WT C. elegans. This work also supports their proposed model of Pf4 expression playing a role in the suppression of PQS quorum sensing and subsequent expression of pyocyanin.

This work was well designed and presented. A major strength of the paper was that it also examined infection from a host perspective, using proteomics and gene ontology to quantify protein expression due to infection and extrapolate possible underlying mechanism. This data is well presented in Figure 4. Their overall proposed model is nicely summarized in Figure 6, which is another major strength of the paper.

We thank the reviewers for the thoughtful summary of our work.

Part II – Major Issues: Key Experiments Required for Acceptance

Reviewer #1: Comments
Could the authors defend why they used rsaL, rhlA and pqsA? Why not lasR or rhlR for

example? I can think of several reasons but it would be good to have the authors state theirs.

We used these previously published quorum sensing (QS) reporters from the Dandekar lab (refs 33-35) because they are promoters from genes known to be regulated by each of the QS transcription factors (LasR regulates *rsaL*, RhlR regulates *rhlA*, and PqsR regulates *pqsA*). As discussed in refs 33-35, *lasR, rhlR*, or *pqsR* expression often does not change significantly when a quorum is achieved, as compared to downstream QS genes such as *rsaL, rhlA*, and *pqsA*. Under the conditions tested, the *rsaL, rhlA*, and *pqsA* reporters are better indicators of QS activation.

I am unsure of the value of the proteomic section. I think the results are well presented and clearly there is a difference between the conditions, I am not sure what value it adds to the publication. It does not spur the work with AhR as far as I can tell. This section could be removed without hurting the conclusions. If I have missed a key connection, please make it more obvious.

We agree that this work could stand alone without the proteomics data. However, a key connection that links the proteomics data to AhR signaling were the five cytochrome P450 proteins (CYP-29a2, CYP-25a2, CYP-14a5, CYP-37a1, and CYP-35b1) that were significantly upregulated in *C. elegans* exposed to ∆Pf4 (Fig 4A, yellow symbols). Because AhR regulates several CYP proteins in animals, the proteomics data strengthens the rationale for pursuing the AhR story. We highlight this link on lines 279-283.

Furthermore, we feel that the *C. elegans* proteomics dataset (Supp Table S1) will be beneficial to the field, a viewpoint echoed by Reviewer #2 in their summary of our work: “A major strength of the paper was that it also examined infection from a host perspective, using proteomics and gene ontology to quantify protein expression due to infection and extrapolate possible underlying mechanism.”

The Pf4 mechanism for interaction with PQS on page 12 could do with some citations as to why the authors think these factors might interact.

Thank you for the comment. In our speculation on page 12 that Pf4 encodes proteins that interact with host PQS signaling proteins (lines 338-343), we cite several studies that characterize phage proteins in other systems that inhibit quorum sensing (Refs 47-50). Of note, *P. aeruginosa* phage LUZ19 encodes a protein called Qst that binds to and inhibits PqsD (Ref 50). We discuss this observation in light of our study and speculate that Pf4 encodes proteins that similarly inhibit PQS signaling. We now discuss how our work adds to a body of literature showing some phages inhibit *P. aeruginosa* QS. We (and others, refs 49 and 50) speculate inhibition of QS makes the host more susceptible

to infection. We have modified this section to better reflect this.

I felt that the mention of medical implants and Cystic fibrosis where a little tacked on. Several of the authors have done fantastic work on QS in CF and Pf4 in CF, I would love to hear their expanded thoughts of how this work might be digested by the fields.

Thank you for the comment. We have expanded our ideas in a revised conclusion paragraph:

“Overall, our study provides evidence that Pf4 phage enhance bacterial fitness against C. elegans predation. Prior work demonstrates that Pf4 phage also enhance bacterial fitness against phagocytes by inhibiting phagocytic uptake [10, 16]. In the environment, nematodes and other bacterivores such as amoeba can impose high selective pressures on bacteria [61-63]. The ability of Pf phage to enhance P. aeruginosa fitness against environmental bacterivores may help explain why Pf prophages are so widespread amongst diverse *P. aeruginosa* strains [3, 64, 65]. The ability of Pf phage to enhance bacterial fitness against bacterivores in the environment may also translate to an increased virulence potential in vertebrate hosts, including humans.”

Finally, I would really like to see the results in figure 5 reproduced with a pyocyanin knockout as well as bacteria load calculations. It would be interesting to know if the bacterial load is constant and the immune response is responding differently. However I understanding asking for additional experimentation can be highly difficult so I am open to arguments.

Thank you for the suggestion. We performed the requested experiments using *P. aeruginosa* Δ*pqsA*, which does not produce pyocyanin (PMID: 17254955). We used Δ*pqsA* in survival assays using wild-type N2 or isogenic AhR-null (*ahr-1(ia3)*) *C. elegans*. The survival curves and median survival data are presented in an updated version of Figure 6 (originally Figure 5), seen below.

Like others before us (PMID 18927620), we find that Δ*pqsA* is far less virulent against *C. elegans* compared to wild-type *P. aeruginosa* (Fig 6A-D, compare black to red). Disabling AhR signaling in *C. elegans* does not significantly affect nematode survival when challenged with Δ*pqsA* (Fig 6C and D). This contrasts with the ΔPf4 mutant where disabling AhR signaling significantly (P=0.0002) increases ΔPf4 virulence compared to wild-type nematodes (Fig 6E and F).

These results indicate that even though pigment production is impaired in Δ*pqsA*, additional virulence determinants are inactivated in the Δ*pqsA* mutant. The results also raise the possibility that the Pf4 prophage is targeted in its inhibition of PQS or other pathways that regulate pigment biosynthesis whose products may be sensed by AhR. We discuss these new findings on lines 287-298.

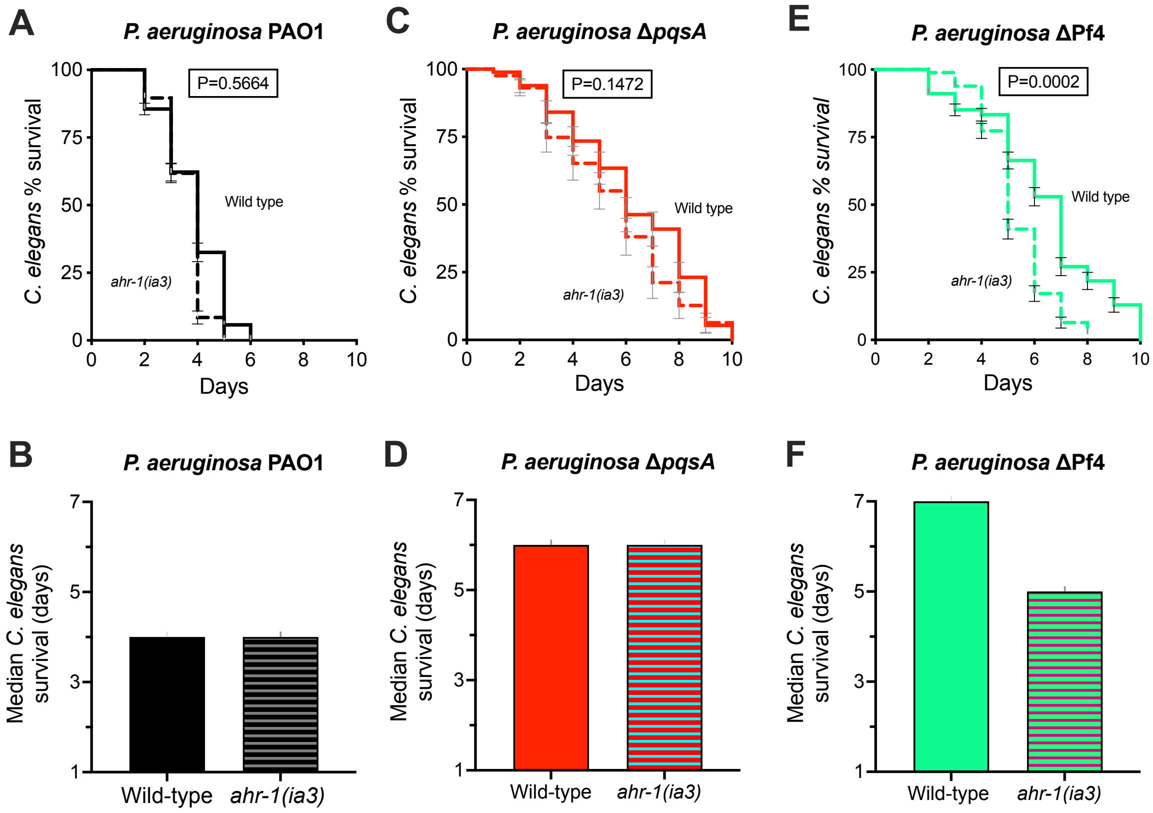

Figure 6. Inactivation of AhR signaling in *C. elegans* enhances ΔPf4 virulence. (A, C, and E) Kaplan-Meier survival curve analysis (Log-rank) of wild-type N2 or isogenic *ahr-1(ia3) C. elegans* maintained on lawns of *P. aeruginosa* PAO1, Δ*pqsA*, or ΔPf4 for the indicated times. N=3 groups of 90 animals per condition (270 animals total per condition). Error bars represent standard error of the mean. P-values of pairwise log-rank survival curve analyses are shown. **(B, D, and F)** The median survival of *C. elegans* in days was plotted for each group.

Reviewer #2: Although the proteomics data is very informative, it would be strengthened further by additional assessment of the cuticle of the C. elegans model. For example, if there is a way to correlate protein abundance (lowered in higher morbidity worms) with actual integrity of the protein. Visual evidence of the breakdown of the cuticle with this prediction would be very useful.

Thank you for the suggestion. Cuticle integrity was assessed by Hoechst staining of nuclei in whole nematodes, as previously described (PMID: 15454573). Briefly, synchronized young adult worms were collected from lawns of PAO1 or ΔPf4 after two days and stained with 10 µg/mL Hoechst for 30 minutes at room temperature. Unbound stain was removed by washing nematodes with M9 buffer before visualization by fluorescence microscopy using a DAPI filter. All nematodes where stained nuclei were observed were scored as permeable and cuticle integrity compromised. We scored 25–50 animals per condition per independent experiment.

We find that cuticle permeability is significantly (P<0.01) increased in *C. elegans*

exposed to *P. aeruginosa* PAO1 compared to nematodes exposed to ∆Pf4 (**Fig 5**). As the referee points out, lower collagen protein abundance in *C. elegans* exposed to PAO1 correlates with loss of cuticle integrity and higher morbidity compared to *C. elegans* exposed to ∆Pf4. We now discuss this on lines 229-238 and we have updated the Methods section (lines 582-590).

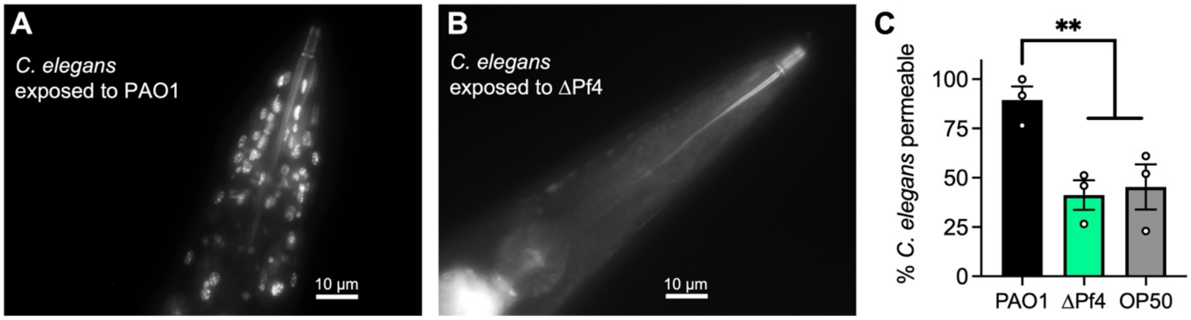

Figure 5. PAO1 compromises *C. elegans* cuticle integrity compared to ∆Pf4. Synchronized young adult N2 worms were collected from lawns of PAO1, ∆Pf4, or *E. coli* OP50 after 48 hours and stained with the nucleic acid stain Hoechst. Cuticle permeability was assessed by visualization of stained nuclei in live nematodes exposed to **(A)** PAO1 or **(B)** ∆Pf4. Representative images are shown. **(C)** The percent *C. elegans* with stained nuclei were scored as permeable and plotted. **P<0.01, Student's *t*-test. N=3 replicates of 25-50 animals per replicate, 92-137 total worms per group.

Part III – Minor Issues: Editorial and Data Presentation Modifications
Please use this section for editorial suggestions as well as relatively minor modifications of existing data that would enhance clarity.

Reviewer #1: Minor mistakes:
Line 117 the words feed on are missing in brackets.

This was changed to "Pf4 cannot infect *E. coli* hosts."

Line 188 Did the authors mean to just write "Methods"?

This change was made.

(very minor) In figures 1-3 the authors use *,**,*** denotations for significance. I'm of the opinion that significance is binary. Being more significant should not be a consideration.

We understand the reviewers' point, however, we respectfully disagree. A p-value measures the probability of obtaining the observed results, assuming that the null

hypothesis is true. This probability is not binary, and we use the following standard convention to provide information about the p-values obtained from our statistical analyses to readers:

ns	P > 0.05
*	P ≤ 0.05
**	P ≤ 0.01
***	P ≤ 0.001

Reviewer #2: Minor revisions:

-Line 97: missing a delta from "In the absence of C. elegans, neither PAO1 nor DPf4 produced any detectable.."

This change was made.

-Line 117: missing "infect" in Pf4 cannot ____ E. coli hosts

This was changed to "Pf4 cannot infect *E. coli* hosts."
